# Performance adaptive training control strategy for recovering wrist movements in stroke patients: a preliminary, feasibility study

**DOI:** 10.1186/1743-0003-6-44

**Published:** 2009-12-07

**Authors:** Lorenzo Masia, Maura Casadio, Psiche Giannoni, Giulio Sandini, Pietro Morasso

**Affiliations:** 1 Robotics Brain and Cognitive Science Dept, Italian Institute of Technology (IIT), Genoa, Italy; 2 Dept of Informatics, Systems and Telematics, University of Genova, Italy; 3 ART Rehabilitation and Educational Center srl, Genoa, Italy

## Abstract

**Background:**

In the last two decades robot training in neuromotor rehabilitation was mainly focused on shoulder-elbow movements. Few devices were designed and clinically tested for training coordinated movements of the wrist, which are crucial for achieving even the basic level of motor competence that is necessary for carrying out ADLs (activities of daily life). Moreover, most systems of robot therapy use point-to-point reaching movements which tend to emphasize the pathological tendency of stroke patients to break down goal-directed movements into a number of jerky sub-movements. For this reason we designed a wrist robot with a range of motion comparable to that of normal subjects and implemented a self-adapting training protocol for tracking smoothly moving targets in order to facilitate the emergence of smoothness in the motor control patterns and maximize the recovery of the normal RoM (range of motion) of the different DoFs (degrees of Freedom).

**Methods:**

The IIT-wrist robot is a 3 DoFs light exoskeleton device, with direct-drive of each DoF and a human-like range of motion for Flexion/Extension (FE), Abduction/Adduction (AA) and Pronation/Supination (PS). Subjects were asked to track a variable-frequency oscillating target using only one wrist DoF at time, in such a way to carry out a progressive splinting therapy. The RoM of each DoF was angularly scanned in a staircase-like fashion, from the "easier" to the "more difficult" angular position. An Adaptive Controller evaluated online performance parameters and modulated both the assistance and the difficulty of the task in order to facilitate smoother and more precise motor command patterns.

**Results:**

Three stroke subjects volunteered to participate in a preliminary test session aimed at verify the acceptability of the device and the feasibility of the designed protocol. All of them were able to perform the required task. The wrist active RoM of motion was evaluated for each patient at the beginning and at the end of the test therapy session and the results suggest a positive trend.

**Conclusion:**

The positive outcomes of the preliminary tests motivate the planning of a clinical trial and provide experimental evidence for defining appropriate inclusion/exclusion criteria.

## Background

Decreased wrist range of motion (ROM) (flexion and/or extension, abduction/adduction or pronation/supination) after trauma or surgery can be a challenging problem. Physical therapy, orthoses, and additional surgical interventions may not restore the desired functionality even after an intensive rehabilitation program. Therapists spend a considerable amount of practice time in differential diagnosis of these losses and selecting appropriate intervention strategies to restore passive and active motion in concordance with the pathology and to prevent loss of range of motion after injury.

While the regular treatment for wrist stiffness is physical therapy or surgery, researchers are looking for an alternative and more efficient and automatic procedure by means of robotic applications.

Several systems for wrist rehabilitation have been developed in research centres and universities, for example RiceWrist [1]; MIME [2]; IMT3 [3], HWARD [4]; the Okayama University pneumatic manipulator [5], and the devices overviewed in [6-9]. The majority are also used for rehabilitation in health centres and hospitals, often coupled with MIT-MANUS [10], ARMIN [11], MIME, HapticMaster [12] and wire-based device from Rosati et. al. [13] for rehabilitation of proximal limb. Robot assisted therapy are primarily based on goal-directed point-to-point movement involving multiple DoFs [14]; main purpose is increasing the ROM of the paretic limb in order to regain motor abilities for the Activities of Daily Living (ADL). Contrarily regular physical therapy of wrist rehabilitation consists in a splinting treatment for each single DoF at time, and there have been many studies that look at the splints' effectiveness and what type of splint would be best [15,16]. Static progressive splinting is a time-honored concept, for more than 20 years, clinicians have recognized the effectiveness of static progressive splints to improve passive range of motion (PROM). Splint designers then sought a means to improve the technique with components that offer infinitely adjustable joint torque control and are easy to apply, lightweight, low-profile, and reasonably priced.

Dynamic splints use some additional component (springs, wires, rubber bands) to mobilize contracted joints [17-19]. This dynamic pull functions to provide a controlled gentle force to the soft tissue over long periods of time, which encourages tissue remodeling without tearing. The issues that make dynamic or static progressive splinting technically difficult include determining how much force to use, how to apply the force, how long to apply the force, and how to prevent added injury to the area. Things could change if the dynamic splinting is delivered using devices which are able to modulate torque delivering and space the range of motion.

Therefore we intend to approach the robotic therapy for wrist rehabilitation using a continuous dynamic splinting of each single DoF but contrarily to the regular progressive splinting we want also to highlight the voluntary component of movement. A performance adaptive control strategy has been developed, with the purpose of providing variable assistance by means of a general training paradigm for stroke patients.

## Methods

### Apparatus: the wrist device

The Wrist-Robot [20], herewith reported, has been developed at the Italian Institute of Technology with three main requirements: 1) back-drivability of the 3 DoFs (Degree of Freedom), in order to assure a smooth haptic interaction between the robot and the patient; 2) mechanical and electronic modularity, in order to facilitate the future integration into a haptic bimanual arm-wrist-hand system with up to 12 DoFs; 3) scalable software architecture. The Wrist Robot is intended to provide kinesthetic feedback during the training of motor skills or rehabilitation of reaching movements. Motivations for application of robot therapy in rehabilitation of neurological patients come from experimental studies about the practice-induced plastic reorganization of the brain in humans and animal models [21,22].

**Figure 1:**
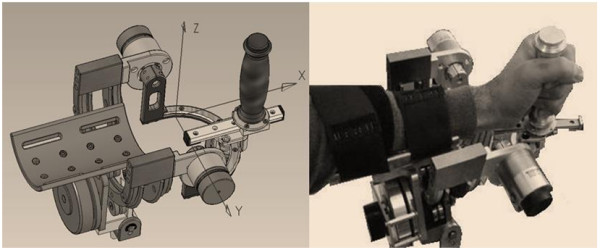
**3DoF Wrist Device**. It has 3 DOFs: F/E, P/S, Ad/Ab. One motor is used for F/E and P/S; two motors for Ad/Ab.

The robot (figure 1) is a 3 DOFs exoskeleton: F/E (Flexion/Extension); Ad/Ab (Adduction/Abduction); P/S (Pronation/Supina-tion).

The chosen class of mechanical solutions is based on a serial structure, with direct drive by the motors: one motor for pronation/supination, one motor for flexion/extension and two parallel coupled motors for abduction/adduction that allow to balance the pronosupination rotation during motion.

The problem of measurement of arm position is thus reduced to the solution of the device kinematics, with no further transformations required, allowing to actuate the robot to control feedback to a specific human joint, for example to constrain the forearm rotation during wrist rehabilitation, without affecting other joints.

The corresponding rotation axes meet at a single point as shown in figure 1.

The subjects hold a handle connected to the robot and their forearms are constrained by velcros^® ^to a rigid holder in such a way that the biomechanical rotation axes are as close as possible to the robot ones. Unavoidable small joints misalignments are partially reduced by means of a sliding connection between the handle and the robot and the forearm can be moved vertically in order to fit the rotation axis of the pronation/supination DoF. In order to minimize the effect of occasional compensatory shoulder/trunk movements during training exercises, the body is firmly strapped to a robust chair and the chair is positioned in such a way to have the elbow flexed about 90 deg and the hand pointing to the centre of a 21" CD screen, in correspondence with the neutral anatomical orientation of the hand.

Having in mind the general requirements of robot therapy [22,23], we identified the following design specifications:

1. sufficient level of the torque at the handle (tab. I)

2. large workspace

low friction and direct drive motors enhance the back-driveability of the manipulandum, thus simplifying its control without needing a closed loop force control scheme. The mechanical range of motion (ROM) is as follows: *F/E *= -70° ↔ +70°; *Ad/Ab *= -35° ↔ +35°; *P/S *= -80° ↔ +80°. These values approximately match the ROM of a typical human subject (Table 1).

**Table 1:** ROM of the Robot and the Human wrist

Wrist Joint	Human joint range of motion [deg]	Wrist Device Workspace Capability [deg]	Human Isometric Torque [Nm]	Wrist Device Continuous torque [Nm]
**Supination/Pronation**	86/71	80/80	5.2	7.1
**Flexion/Extension**	73/71	70/70	19.8	12.4
**Abduction/Adduction**	33/19	35/35	20.8	12.9

comparison between range of motion and joint torque of a human [24-26] and the IIT-wrist device; the values of the continuous delivered torque are obtained by a design compromise between backdrivability and power requirements based on anthropometric data.

Each DOF is measured by means of a high-resolution encoder (2048 bits/rev) and is actuated by one or two brushless motors, in a direct-drive, back-drivable connection, providing the continuous torque values reported in table 1. The control architecture integrates the wrist controller with a bi-dimensional visual virtual reality environment (VR) for showing to the subjects the actual joint rotation transformation of the hand, the corresponding target direction and two performance indicators defined in the following. The software environment is based on Simulink^® ^and RT-Lab^®^. The control architecture includes three nested control loops: 1) an inner loop, running at 7 kHz, used by the motor servos; 2) an intermediate loop, running at 1 kHz, for the low level control; 3) a slower loop, running at 100 Hz, for implementing the VR environment and the user interface. The mechanical structure of the wrist robot was designed in such a way to allow a simple and immediate mounting for patients' forearm.

### Task

The task is mono-dimensional tracking of a sinusoidally moving target, using one DOF at a time: F/E, Ad/Ab or P/S, respectively; this approach is consistent with the dynamic splinting paradigm which is primarily used to regain the passive ROM after trauma or surgical intervention; the subject aims to move the handle to track the harmonic motion of the target using his/her active ROM; the robot gently intervenes if the subject is not able to actively cover the required angular displacement. Three different experiments were then carried out for the three different DoFs of the wrist. For each experiment, there was one active DoF, which received controlled assistance by the robot, while the two other DoFs were hold by the robot in a small neighbourhood of the neutral position [24-26].

In order to make the task interesting and challenging at the same time, the level of difficulty was managed by the controller modulating two parameters as a function of the performance: a) frequency of the target motion; b) level of the robot assistance. The controller implementation is discussed and illustrated in the next section.

### Controller architecture

The general control architecture consists of three blocks: 1) target motion generator; 2) force filed generator; 3) performance evaluator.

**Figure 2:**
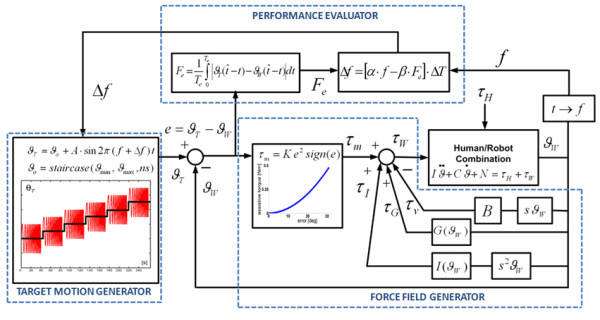
**Controller diagram**. The "assist-as-needed" force parabolic term continuously inputs torque *τ*_*m *_when errors are present during the tracking task. The input torque to the robot/hand system is the sum of different contributions of a viscous field *τ*_*v*_, a gravity *τ*_*G *_and inertia *τ*_*I *_compensation. *τ*_*H *_is the torque applied by the subjects wrist.

Figure 2 shows (on the left) the control scheme named "*Target Motion generator*" and exemplifies a segment of the oscillatory pattern that span the entire ROM in a progressive manner. The *Target Motion Generator *is characterized by the following set of equations that are sampled at 1 kHz by the inner control loop and they will be explained in present section.

Here *ϑ*_*W *_stands for the joint angular rotation of anyone of the three DoFs of the robot: F/E, Ab/Ad, P/S (figure 2). In particular, *ϑ*_*T *_is the time-varying target angular position, characterized by an harmonic motion with frequency *f*, amplitude *A*, and bias or offset *ϑ*_*o *_(eq. 1).(1)

The bias is moved in a staircase manner (eq. 2), in order to progressively span the whole ROM of each DoF (*ϑ*_min _↔ *ϑ*_max_) by means of *ns *steps (*ns *= 11 in our experiments).(2)

Each step of the staircase has a duration of 40s plus a 4s rest interval, during which the harmonic motion of the target is stopped as well as the attractive force. For each DoF, the ROM is scanned by the staircase starting from the "easier" to the "more difficult" angular position, taking into account the specific pathological conditions of the treated subjects. In this feasibility study the sequence was, for all the patients, from Flexion to Extension, from Adduction to Abduction, and from Pronation to Supination, respectively. The sequence is ordered "from easy to difficult" considering the hypertonic trend in the range of motion for each trained DoF: 1) the offset angle steps from the easy (more natural and less hypertonic) to the difficult (less natural) joint configuration; 2) the oscillation is modulated from slow (easy) to quick (difficult) frequency.

Table 2 shows the amplitude of the target oscillations and the range of values of the angular offset/bias: such range is divided into 11 parts corresponding to the steps of the staircase. Therefore each step amplitude is different for the different three spaced ROMs. Thus, the subjects are progressively trained in a limited workspace but the gradual change of the offset angle allows them to experience the whole ROM for each single DoF (as a progressive splinting). The initial position was chosen taking into consideration the specific pathological conditions; i.e. subjects train each Dof starting form the less hypertonic portion of each ROM to gradually space the whole workspace.

**Table 2:** Growth and decay coefficients of Eq. 9 for each DOF and amplitude oscillation and max/min ROM for each Dof

Joint	α [Hz]	β [Hz^2^/rad]	A [deg]	*ϑ*_min _[deg]	*ϑ*_max _[deg]
**FE**	0.2	0.0012	11.5	-14.5	14.5
**AA**	0.2	0.0015	11.5	-14.5	14.5
**PS**	0.25	0.0008	14.5	-30.5	30.5

The table provides the growth (α) and decay coefficient (β) used by the performance evaluator block of the controller to change the frequency of oscillation of the target. For each DOF, the table stores the amplitude (A) of the target oscillations while *ϑ*_min_, *ϑ*_max _are the minimum and maximum value assumed by the angular offset *ϑ*_*o *_to space the range of motion of each Dof.

Eq. 3 identifies the tracking error for each time instant (*ϑ*_*w *_is the current angular position of the wrist DoF) which is input in the *"Force Field Generator" *and the "*Performance Evaluator*".(3)

The assistive torque provided by the motor is computed in the *"Force Field Generator" *according to eq. 4 and then transformed into the corresponding current drive.(4)

The actual delivered torque *τ*_*w *_is the sum of different control efforts that consider assistance *τ*_*m *_(eq. 5), gravity compensation *τ*_*G *_(eq. 6), inertia compensation *τ*_*r *_(eq. 7) and a viscous field *τ*_*v *_(eq. 8) in order to stabilize by a damping effect the unwanted oscillation at the end effector.(5)

The different contribution of the force field generator is shown in figure 2 (right).

The assistive control law *τ*_*m *_consists of a non linear elastic field with a parabolic profile (eq. 5). This non linear characteristic was chosen according to the principle of *minimal assistance *[27] or also *assist as needed *[28]: assistance forces/torques should be kept as low as possible in order to promote the emergence of voluntary control. In fact, the chosen pattern of assistance has a less-than-linear increase for small errors, thus facilitating the emergence of active un-aided control at the end of training; for large errors, which are likely to occur at the beginning of training, the assistance grows more than linearly in order to speed up the learning process. The same concept of minimal assistance is used for selecting, in an individual-specific manner, the gain ***K***: it is chosen as the minimum value capable to induce the initiation of movements of the paretic wrist and it was chosen by experimentally observing the active voluntary movements of the participating subjects before starting the rehabilitation protocol.

The "*Performance Evaluator" *computes intermittently the average angular error given by eq. 3 in a time window (*T*_*e *_= 2 s):(9)

where  is the time instant at which the current oscillation terminates or also the zero-crossing of the *ϑ*_*T*_-*ϑ*_*W *_waveform.

The "*Performance Evaluator" *modulates the "difficulty" of the tracking task, i.e. the oscillation frequency *f *= 1/Δ*T*, by changing it in a smooth way at the end of each complete oscillation cycle according to the following equation:(10)

The equation contains two terms: a raising term with a coefficient *α *and a decaying term depending on the average angular error *F*_*e *_multiplied by the decay coefficient *β*. For clarity sake figure 2 shown the entire controller scheme highlighting the different blocks of the controller. There are also two saturation levels that keep the task in a suitable range of difficulty: we chose the range 0.1-1.0 Hz empirically, looking at the performance of the unimpaired subjects. Also the values of *α *and *β *for each DoF were experimentally chosen, in order to balance the conflicting requirements of *readiness *and *smoothness *and provide a symmetric counterbalance of decaying and raising contributions: these values are listed in table 2.

During the performance of an exercise, when eq. 2 switches the offset *ϑ*_*o *_from one step to the next one, the initial value of eq. 10 is reset to the minimum value of frequency (0.1 Hz). Therefore, the initial target oscillation will be very slow and will smoothly speed-up as a function of the tracking accuracy *e *= *ϑ*_*T *_- *ϑ*_*W*_, until the end of the step (40s).

### Virtual Reality environment

**Figure 3:**
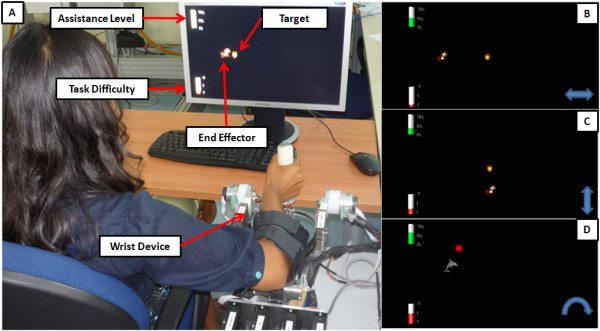
**Virtual reality environment in the therapy session**. A) Experimental set-up in the P/S case: the dolphin chasing the ball. The two bars on the left of the screen display two performance indicators. B) F/E excercise; D) Ab/Ad excercise; D) P/S exercise.

The VR process displays on the screen the trajectory of the target and the wrist angular position (figure 3). The target and the wrist positions are represented graphically as 'pleasant' images: a *dolphin *chasing a *ball *or a *squirrel *hunting an *acorn*. The target path on the PC screen is horizontal in the F/E experiment, vertical in the Ab/Ad experiment, and a circular segment in the P/S experiment.

We wanted to strengthen the effectiveness of the system in monitoring wrist use while providing encouragement and reminders throughout a therapy session [29].

Hence we also display, on the left side of the screen, the instantaneous levels of the two performance indicators by means of height-modulated bars: 1) the level of assistance and 2) the frequency of oscillation. The patients were instructed to minimize the height of the former one while maximizing the height of the latter. This kind of intuitive performance feedback was easily understood by the patients and well appreciated by them.

### Subjects

Three stroke subjects volunteered to participate in this preliminary study. The recruitment was among the outpatients of the ART Rehabilitation and Educational Centre (Genoa, Italy), and based the following inclusion criteria: 1) diagnosis of a single, unilateral stroke verified by brain imaging; 2) sufficient cognitive and language abilities to understand and follow instructions; 3) chronic condition (at least 1 year after stroke). Table 3 summarizes the anagraphic data (age, sex) and the clinical state (etiology, disease duration, affected side, Fugl Meyer and Ashworth scores) collected at the ART Rehabilitation and Educational Centre (Genoa, Italy). The research conforms to the ethical standards laid down in the 1964 Declaration of Helsinki, which protects research subjects. Each subject signed a consent form that conforms to these guidelines. The robot training sessions were carried out at the Human Behaviour Lab of IIT (Genoa, Italy), under the supervision of an experienced physiotherapist of the ART Rehabilitation and Educational Center.

**Table 3:** Patients demographics

ID	Age	Sex	DD	Eti	PH	FM	Ash
S1	37	F	5	I	R	25	1+

S2	57	F	3	H	L	36	1

S3	60	M	6	H	L	22	3

Age & DD (disease duration): years; Eti (etiology): Ischemic/Hemorrhagic; FM: Fugl-Meyer score (arm section 0-66); Ash: Ashworth score (0-4). PH: paretic hand (Right/Left).

### Collected Data

The following parameters were estimated for each DoF:

- Max frequency: the maximal frequency that the subject is able to reach, in the possible range 0.1-1 Hz;

- Mean assistive torque: the average torque delivered to the patient during the rehabilitation protocol for each DoF

- ROM achieved in the single step;

- Mean speed.

Moreover we estimated:

- The ROM in the whole session (minimum-maximum degree of movement in the entire exercise).

- The active voluntary ROM of the subject holding the passive inactivated device, before and after the exercise in order to compare if the rehabilitation protocol would provide fast benefits even after one therapy session.

## Results

**Figure 4:**
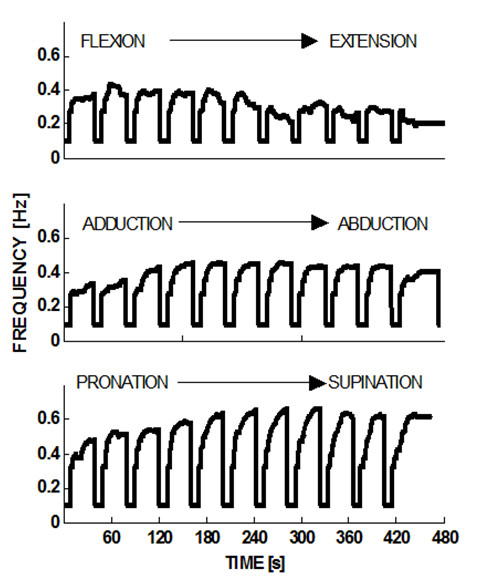
**Course of the target frequency when the offset position steps through the ROM**. At the beginning of each step the frequency is reset to its minimum value (0.1 Hz); the maximum possible value is 1 Hz. Subject S3.

Although the clinical states of the three subjects are rather different, as reported in table 3, all of them were able to carry out the proposed exercises in a consistent way, with different performance profiles considering the performance adaptive nature of the controller architecture. For clarity sake, in the present preliminary/feasibility study, the following figures will refer to subject S3, who is the most severely affected and therefore the worst case in the experienced population. Figure 4 shows the evolution of the frequency of the moving target for each DoF, while the *ϑ*_*o *_position scans through the 11 values that are uniformly placed in the corresponding ROM: 40s for each step + 4s of rest between one step and the next one. For each step, the peak value of the frequency depends on the position in the workspace of each DoF and on the specific pathological condition of each patient: the figure shows that S3 has higher difficulty in extension than flexion, in adduction than abduction, and in pronation than supination.

**Figure 5:**
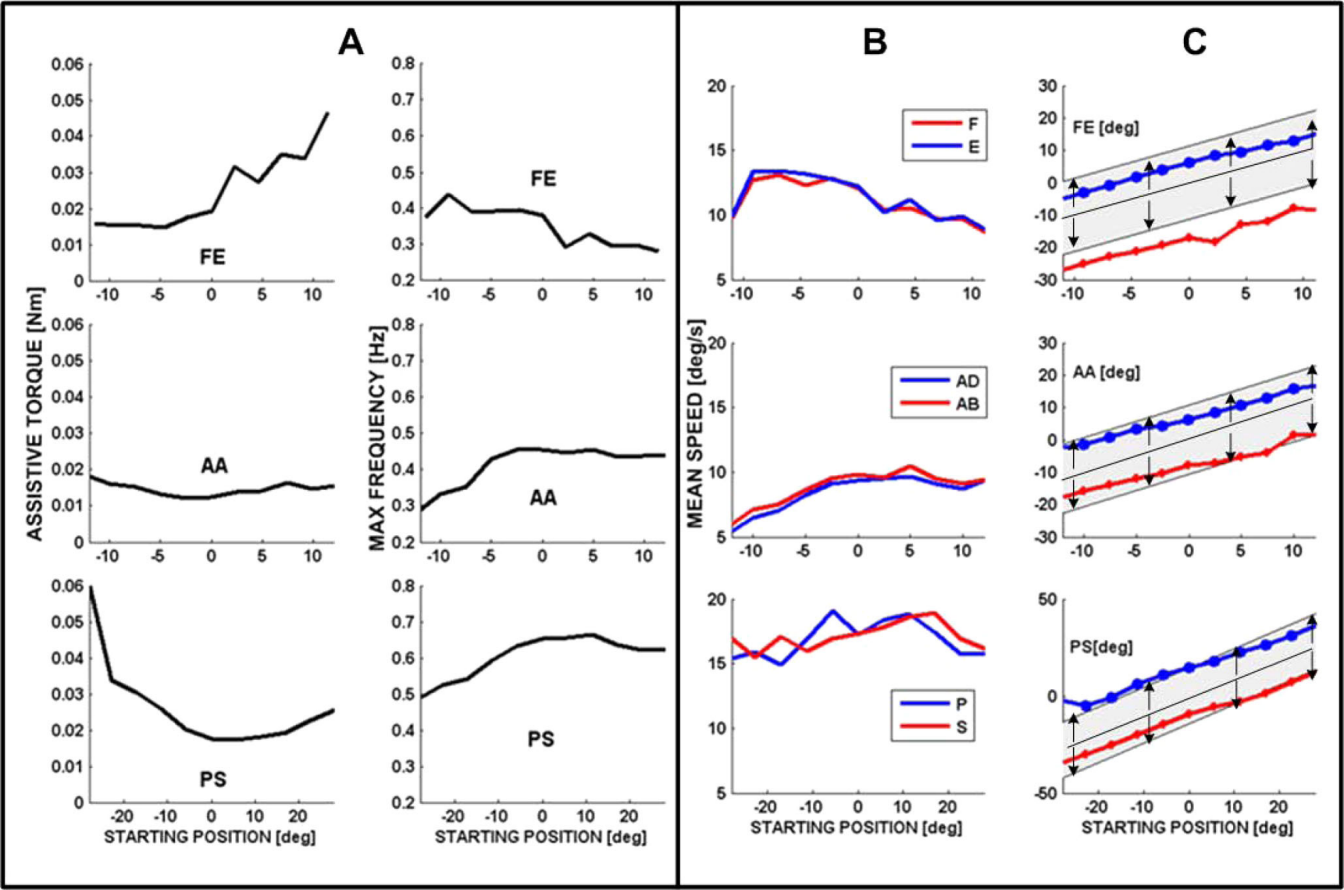
**complementary analysis between assistive torque and maximum frequency reached during tracking (subject S3)**. (A) Left panel: Maximal target frequency reached for the different DOFs during the 40s steps, identified by the starting position in the ROM with respect to the neutral position. Right panel: Mean value of the assistive torque (in 10-3Nm) during the corresponding steps.(B) Mean tracking speed, for the different DOFs, in the different 40s steps, identified by the starting position in the ROM with respect to the neutral position. Gray and black curves correspond to the opposing parts of the movements (F vs. E, Ad vs. Ab, P vs. S). (C) For each value of the offset rotation and each DOF, the graphs show the ROM of the robot (shaded band) and the ROM of subject S3 (black curves). X-axis identified the spammed ROM for the exercised Dof; positive and negative value are referred respectively to F/E, Ab/Ad and P/S while zero is the neutral position. Y-axis is the amplitude oscillation reached by the target (shaded band) and by the subject.

Figure 5A summarizes the trend of the peak frequency at the different steps comparing it with the corresponding evolution of the assistive torque provided by the robot. It appears that the two sets of curves provide compatible and complementary messages as regards the overall performance of S3: he reaches peak frequency at about full flexion and mid-range of abduction/adduction and prono/supination; in the same areas the assistance torque reaches local minima, highlighting the fact that higher performance is obtained when a higher capability of voluntary motion is present needing a lower level of assistance.

The information provided by figures 4 and 5A is complemented by the measurement of the Active ROM (voluntary capability of moving) for each type of movement of the wrist DoFs. These measurements were carried out at the beginning and at the end of the training session, by using the same wrist robot in order to normalize the intrinsic constraints (biomechanical and neurological) as well as the constraints determined by the robot. In the measurement, only one DoF at a time was allowed to move freely (with no assistive control applied), while the two remaining DoF were hold by the robot in the approximated neutral positions. Table 4 summarizes the measurements before starting the protocol. Shaded cells correspond to the more impaired movements for each subject: 1) all of them lack mobility in Extension rather than Flexion; 2) S1 has a higher deficiency in Abduction that in Adduction, while S2 and S3 have the opposite impairment; 3) S1 shows a higher deficit in Supination whereas S2 and S3 are worse in Pronation.

**Table 4:** Active Range of motion of the subjects pre and post treatment

PRE-TREATMENT							
**ID**	**F** **[deg]**	**E** **[deg]**	**AD** **[deg]**	**AB** **[deg]**		**P** **[deg]**	**S** **[deg]**
**S1**	60	**7.5**	12.2	**3.0**		6.0	**3.8**
**S2**	61.5	**3.1**	**23.0**	21.7		**12.5**	23
**S3**	59.5	**-8.4**	**10.5**	28.4		**6.0**	21.5

**POST-TREATMENT**							

**ID**	**F** **[deg]**	**E** **[deg]**	**AD** **[deg]**	**AB** **[deg]**	**P** **[deg]**		**S** **[deg]**
**S1**	25.67	**19.23**	18.62	**15.68**	38.90		**37.41**
**S2**	28.49	**19.15**	**16.01**	22.33	**37.59**		36.91
**S3**	27.38	**15.70**	**18.24**	19.22	**34.88**		36.67

(A) Active voluntary range of motion measured using the uncontrolled (not active) device before treatment. Grey cells correspond to the more difficult movements for the subjects. (B) Reached ROM evaluated during treatment. Bold data correspond to the more impaired movements for the subjects considering each pathological condition.

A similar kind of pattern, i.e. asymmetry of performance for easier vs. more difficult movement directions, can be shown as regards the maximal values of frequency reached by the target (table 5).

**Table 5:** Maximal frequency reached and average assistive torque

MAXIMAL FREQUENCY REACHED						
**ID**	**F** **[Hz]**	**E** **[Hz]**	**AD** **[Hz]**	**AB** **[Hz]**	**P** **[Hz]**	**S** **[Hz]**
**S1**	0.45	**0.35**	0.51	**0.38**	0.75	**0.57**
**S2**	0.59	**0.37**	**0.31**	0.53	**0.52**	0.71
**S3**	0.44	**0.28**	**0.28**	0.45	**0.48**	0.66

**AVERAGE ASSISTIVE TORQUE**						

**ID**	**F [mNm]**	**E** **[mNm]**	**AD** **[mNm]**	**AB** **[mNm]**	**P** **[mNm]**	**S** **[mNm]**
**S1**	12	**11**	9	**16**	15	**27**
**S2**	12	**12**	**8**	6	**26**	19
**S3**	15	**32**	**15**	16	**32**	20

Maximum value of frequency oscillation reached by the subjects for each type of exercised Dof direction during robot training. Average assistive torque required by each subject for the extreme values of each type of motion (e.g. maximum Flexion, etc.). Bold numbers cells indicate more impaired movements.

We can also observe that minimal frequency values correspond to the position in which subjects have a reduced range of motion. Moreover, table 5 shows that maximal assistive joint torque is generally provided on the side of the movement of each DoF where the subject is more defective.

The performance of the subjects can also be investigated by comparing the mean speed of the two opposite movements for each DoF in relation with each offset step of the staircase (Figure 5B: F vs. E, Ad vs. Ab, and P vs. S).

We can observe that, for each DoF, the speed curves for the opposing rotations are quite similar in spite of the fact that there is a significant asymmetry in the ROM, as shown in tables 4 before and after threatment. This suggests that the training protocol is effective in two main ways, by inducing at the same time the patient to behave in a more functional and physiological way:

1) exercising movements that are more difficult for him/her, given his specific pathological condition, for example Extension vs. Flexion;

2) moderating the predominance of pathology-aided behaviours that would enhance Flexion vs. Extension etc.

At last, figure 5C compares, for each DoF, the ROM of the robot target motions (shaded grey band is the amplitude of the target oscillation at different starting position on each DoF workspace) with the actual ROM (bold lines with markers for the two directions of each Dof) exhibited by patient S3 in relation with each offset position. It appears that generally the maximal joint rotation achieved by the patient is asymmetric in the two opposing directions of each DoF (P vs S, F vs. E, Ad vs. Ab) and this is reflected in the pattern of values stored in table 4 of the active range of motion measured by the uncontrolled device at the beginning of protocol. i.e. In spite of the assistance, the subject S3 does not succeed in following the harmonic motion of the target represented by the shaded grey band; he systematically undershoots extension (blue line) and overshoots flexion (red line), whereas the performance is closer to physiological conditions for the two other DoFs.

On the other hand, table 4 reports the active range of motion (uncontrolled device) measured at the end of the training session and the comparison between the part of the table 4 shows a clear increase and symmetrisation before and after the threatment; this result suggests that using robot to generate mobilising splints might be useful to modify the joint stiffness, and reducing hypetonia; even if the total ROM is reduced the symmetry noticeably increases; it is possible the passive component due to hyper tonicity before the splinting added a bias to each joint drifting from the anatomical neutral position.

In the lights of these considerations however we present a preliminary study on the feasibility of using a performance adaptive control strategy combined with a dynamic splinting; in order to strengthen the effectiveness of the proposed approach a wider clinical protocol with higher number of subjects and therapy session is needed.

## Discussion

Although it has been shown in a number of studies that robots can decrease motor impairment after stroke with certain advantages, less emphasis to date has been put on robotic developments for the hand and on corresponding preliminary clinical studies. A notable exception is the work by Takahashi et al. [4] who reported the use of the pneumatic-actuated HWARD wrist robot with 13 patients. The main difference of HWARD with respect to the Wrist robot (here with reported) is related to the wrist movements: HWARD can only operate with F/E whereas Wrist Robot can operate equally well with Ab/Ad and P/S.

In this preliminary experiment investigating patients, only one joint DoF was exercised at a time. The procedure simulated as much as possible the use of splints widely used in clinical applications. However, there is no hardware or software limitation to design 2D and 3D experiments, which indeed are planned and will be carried out in the near future.

We wish to emphasize that our control system is based of a principle of minimal assistance that focuses on the initiation of the movement; on the contrary most of the other rehabilitation robots, focuses on the termination phase (goal directed movements), by forcing the patient to complete the movements if he/she is unable to achieve the target. We also plan to integrate in the robot an active finger F/E unit, by means of a motorized handle [30] to study the impact of single-DoF rehabilitation protocol on cylindrical grasping and compare the effectiveness of different rehabilitation strategies that include distal and/or proximal limb.

The results reported in this single-session study show that the proposed adaptive control strategy is robust, in terms of patient response, is well accepted by the subjects and the control architecture is capable to smoothly adapt to the specific impairments of the patients without needing a fine customization of the controller gains for each subject; this controller robustness allows to introduce the system in the clinical application providing a user friendly interface for users and patients, and to deliver an automatic execution of the therapy sessions.

## Conclusion

The results of the presented preliminary work shows that robotic therapy may improve motivations in patients and provide tangible results even in a short term experience. The technological approach with the use of customized devices may strengthen the potentials of the regular physical therapy in delivering assistance and training. The proposed controller strategy is simply based on an automation of the well established methodology of dynamic splinting; this kind of approach can result familiar to the medical staff allowing technology to progressively take part to the emerging and increasing needs of rehabilitation, without shocking the entrenched application of regular therapy. It remains to be investigated, as we plan to do in a systematic clinical trial, to which extent a suitable protocol can induce permanent improvements in the neural control of wrist movements, necessary for any attempt to achieve functional gains in the activities of daily life.

## Competing interests

The authors have not competing interests as defined by the BioMed Central Publishing Group, or other interests that may influence results and discussion reported in this study.

## Authors' contributions

LM conceived and designed the device used in the present work. LM and MC carried out the experiments and the data analysis and drafted the manuscript; PM participated in the design of the study and carried out the experiment; PG participated in the coordination of the study and conceived the rehabilitation protocol, assisting the patients during the robot therapy sessions; GS conceived of the study, and participated in its design and coordination.

All authors read and approved the final manuscript.
